# Fecal and Tissue Microbiota Are Associated with Tumor T-Cell Infiltration and Mesenteric Lymph Node Involvement in Colorectal Cancer

**DOI:** 10.3390/nu15020316

**Published:** 2023-01-09

**Authors:** Kayti Luu, Jason Y. Ye, Venu Lagishetty, Fengting Liang, Megan Hauer, Farzaneh Sedighian, Mary R. Kwaan, Kevork K. Kazanjian, J. Randolph Hecht, Anne Y. Lin, Jonathan P. Jacobs

**Affiliations:** 1 John A. Burns School of Medicine, University of Hawai’i at Mānoa, Honolulu, HI 96813, USA; 2 The Vatche and Tamar Manoukian Division of Digestive Diseases, Department of Medicine, David Geffen School of Medicine at UCLA, Los Angeles, CA 90095, USA; 3 Division of Gastroenterology, Hepatology and Parenteral Nutrition, Veterans Affairs Greater Los Angeles Healthcare System, Los Angeles, CA 90073, USA; 4 Division of General Surgery, Department of Surgery, David Geffen School of Medicine at UCLA, Los Angeles, CA 90095, USA; 5 Division of Hematology/Oncology, Department of Medicine, David Geffen School of Medicine at UCLA, Los Angeles, CA 90095, USA

**Keywords:** tissue-associated microbiome, colorectal cancer, mesenteric lymph node involvement, tumor T cell infiltration, biomarkers

## Abstract

Colorectal cancer (CRC) is associated with alterations of the fecal and tissue-associated microbiome. Preclinical models support a pathogenic role of the microbiome in CRC, including in promoting metastasis and modulating antitumor immune responses. To investigate whether the microbiome is associated with lymph node metastasis and T cell infiltration in human CRC, we performed 16S rRNA gene sequencing of feces, tumor core, tumor surface, and healthy adjacent tissue collected from 34 CRC patients undergoing surgery (28 fecal samples and 39 tissue samples). Tissue microbiome profiles—including increased *Fusobacterium*—were significantly associated with mesenteric lymph node (MLN) involvement. Fecal microbes were also associated with MLN involvement and accurately classified CRC patients into those with or without MLN involvement. Tumor T cell infiltration was assessed by immunohistochemical staining of CD3 and CD8 in tumor tissue sections. Tumor core microbiota, including members of the *Blautia* and *Faecalibacterium* genera, were significantly associated with tumor T cell infiltration. Abundance of specific fecal microbes including a member of the *Roseburia* genus predicted high vs. low total and cytotoxic T cell infiltration in random forests classifiers. These findings support a link between the microbiome and antitumor immune responses that may influence prognosis of locally advanced CRC.

## 1. Introduction

Despite the widespread implementation of colorectal cancer (CRC) screening, CRC remains the third most commonly diagnosed cancer and the third leading cause of cancer death for both men and women in the United States [1,2,3]. CRC detected at locally advanced stages (II and III) can potentially be cured by surgical resection; however, the risk of recurrence is high due to micrometastasis. While the addition of adjuvant chemotherapy treatment has helped reduce three-year recurrence rates, there is still a 20–30% risk of recurrence [4,5]. Given that CRC continues to be a significant cause of morbidity and mortality, there is a need to understand the environmental factors contributing to the progression of CRC and influencing the likelihood of recurrence after surgery and adjuvant therapy.

The intestinal microbiome is a key environmental factor in the development of CRC [6,7,8]. Previous animal models studying CRC have shown invasive microbes such as *Fusobacterium nucleatum* and a tumor-associated *E. coli* strain to directly promote intestinal adenoma formation [9,10]. Furthermore, human studies have consistently identified distinct microbiota, particularly *Fusobacterium* and invasive *Escherichia coli*, in the stool and tissue of patients with CRC and adenomas compared to healthy controls [7,11,12,13].

The intestinal microbiome not only affects the development of CRC, but also plays a role in the outcome of immunotherapy and chemotherapy. Multiple animal studies have found that the presence of the intestinal microbiome is required to trigger appropriate antitumor immune responses [14,15,16,17]. Animal models also found certain microbes to be associated with either resistance or response to antitumor therapy [15,18,19]. High profile studies have extended these findings to humans, demonstrating that the gut microbiome influences the response to chemotherapy and immunotherapy through effects on antitumor immune responses [18,19].

In locally advanced CRC patients, survival after surgical resection correlates with the degree of tumor infiltration by T cells, particularly CD8+ cytotoxic T cells and memory T cells [20,21]. Given prior preclinical studies demonstrating that the microbiome can modulate T cell infiltration into CRC [15,22,23] and also influence CRC metastasis [24,25], we hypothesized that the intestinal microbiome would be associated with variation in degree of T cell infiltration and mesenteric lymph node involvement in CRC patients. Of note, a prior 16S rRNA gene sequencing study of 27 tumor samples reported differential abundance of multiple bacterial taxa between CRC patients with high vs. low T cell infiltration [23]. Associations have also been reported between tumor-associated microbes and levels of T cell attracting chemokines [23,26]. The small sample size and lack of fecal microbiome profiling in the prior study supported the need to validate the findings and further investigate the relationship of the microbiome with tumor T cell infiltration in human CRC. In this study, we characterized associations of fecal and tissue microbial profiles of CRC patients with total and cytotoxic T cell infiltration as well as with tumor mesenteric lymph node involvement. 

## 2. Materials and Methods

Patient Recruitment: Subjects were recruited at the University of California, Los Angeles (UCLA) Jonsson Comprehensive Cancer Center during regularly scheduled clinic visits in the colorectal surgery clinics (demographic traits shown in Table 1). Inclusion criteria included men and women over 18 years of age with colorectal adenocarcinoma who had been deemed candidates for surgical resection. Rectal cancer patients who received neoadjuvant radiation therapy were included. Patients with history of malignancy other than colorectal adenocarcinoma within the past 5 years, history of inflammatory/autoimmune disorders (e.g., inflammatory bowel disease, rheumatoid arthritis, psoriasis, etc.), and who were receiving treatment with medications that affected immune function were excluded. All subjects signed the UCLA Institutional Review Board-approved written informed consent prior to the initiation of any study specific procedures. Voluntary informed consent for participation in this study and processing of personal data was obtained from all subjects involved in the study.

Clinical Metadata Extraction: Clinical data extracted from the electronic medical record included age, comorbidities, medications, tumor location, and tumor stage. After surgery, the local tumor extent, mesenteric lymph nodes, lymphovascular invasion, and histologic grade were also extracted from the electronic medical record.

Fecal Sampling: Fecal samples were collected prior to surgery. Subjects were provided with stool kits for home sampling. Subjects transferred freshly defecated feces to Para-Pak collection vials containing 95% ethanol to fix samples which allowed metagenomics profiles to be stable at room temperature for up to 2–4 weeks [27]. Samples were then mailed to UCLA and upon arrival were immediately stored in −80 °C until processing. 

Tissue Sampling: Tissue was collected by the UCLA Translational Pathology Core Laboratory (TPCL) after surgical removal of the primary tumor site. Upon dissection in the pathology suite, 1 g pieces of the luminal portion of the tumor, the core of the tumor, and healthy adjacent mucosa were cut. The tissue samples were placed in cryovials then flash frozen with liquid nitrogen and stored at −80 °C.

Immunophenotyping of Tumor Tissue: Sections of formalin-fixed, paraffin-embedded primary tumor were obtained from the UCLA TPCL and stained with antibodies against CD3 and CD8. The number of stained cells per mm^2^ was then counted using ImageJ. This was accomplished through deconvoluting the hematoxylin, DAB chromogen, and eosin stained image color plane from histology images and thresholding the stained cells from the background. Cell counting was refined by implementing FFT bandpass filtering, which eliminated stray pixels and small image features. Beyond automated cell-counting, the background was thresholded similarly and scaled to determine the precise area of the tissue within the image for the calculation of density. Dichotomized high vs. low T cell infiltration categories were determined using cutoffs adapted from prior studies on T cell CRC infiltration and survival [20,21]. Histology was unavailable for some rectal cancer patients with robust response to neoadjuvant radiation.

16s rRNA Gene Sequencing: DNA was extracted from fecal samples using bead beating in conjunction with the Qiagen Powersoil kit. Sequencing of the V4 region of 16S ribosomal DNA was performed by the UCLA Microbiome Core as previously described by Illumina MiSeq v2 kit (2 × 250 bp) [28]. Raw sequence data were processed in QIIME using DADA2 and matched to the Silva database v138.1 to identify amplicon sequence variants (ASVs)—roughly corresponding to species [29,30]. After processing, the sequence depth ranged from 4497 to 58,644, with a mean of 30,724 sequences per sample.

Bioinformatics Analysis: Microbial alpha diversity was calculated for each sample using Chao1 and Shannon index on data rarefied to 4497 sequences per sample. Linear mixed effects models were used to assess the significance of the relationship of microbial alpha diversity in tissue samples to tumor T cell infiltration, tumor stage, mesenteric node presence, age, and sex, adjusting for subject as a random effect. Analysis of variance was used for fecal samples. Microbial composition was compared across all samples (beta diversity analysis) using Bray–Curtis dissimilarity and visualized with principal coordinates analysis. Significance of differences in microbial composition by tumor stage, mesenteric node presence, total T cell infiltration (CD3), and cytotoxic T cell infiltration (CD8) was determined using permutational multivariate analysis of variance (PERMANOVA) implemented in the Adonis function of the R package vegan [31]. Repeated measures aware PERMANOVA was used for tissue samples [32].

MaAsLin 2 (Microbiome Multivariable Associations with Linear Models) was used to fit ASV and phylum abundances to generalized linear and mixed effect models while adjusting for covariates and subject as a random effect for tissue samples [33]. Estimates of significance were adjusted for multiple hypothesis testing to generate q-values [34].

Differentially abundant ASVs were used to construct random forest classifiers in the R package caret for tumor phenotypes of interest, in particular, the degree of CD8+ and CD3+ T cell infiltration (high/low) [35]. Specificity and sensitivity of the classifiers for the dichotomized categorical variables were used to generate ROC curves. Importance scores, representing the contribution of an ASV to classifier accuracy, were calculated for each bacterial ASV included in the classifiers.

## 3. Results

### 3.1. Tumor and Healthy Adjacent Tissue Samples from Colorectal Cancer Patients Show Distinct Microbiome Profiles Compared to Fecal Samples

Thirty-four colorectal cancer (CRC) patients were recruited from colorectal surgery clinic for collection of pre-operative stool samples and tumor tissue upon surgical resection. Most subjects (85%) had left-sided CRC, with 32% having involvement only of the rectum, 15% of the rectosigmoid junction, and 38% of the sigmoid colon (Table 1). The remaining subjects had involvement of the transverse colon (12%) and ascending colon (3%). Fecal samples were obtained from 28 subjects (the remaining 6 were not able to submit stool prior to surgery) and tissue samples were collected from 13 subjects. Three tissue samples were collected from each subject, including the center of the tumor (core), the surface of the tumor facing into the colonic lumen, and healthy adjacent colonic tissue. Tissue was not available from the remaining subjects due to inadequate material (e.g., small tumors for which all tumor tissue was required for clinical purposes) or lack of staff availability to perform tissue collection on the day of surgery. 

**Figure 1:**
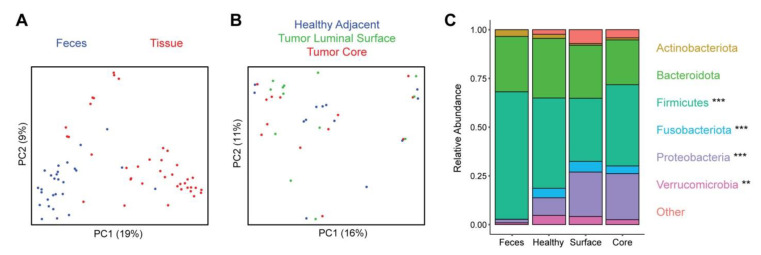
Distinct microbiota composition between feces and tissue sampling sites. (**A**) Microbial composition of fecal and tissue samples was visualized by principal coordinates analysis (PCoA). Each dot represents one sample, with fecal samples in blue and tissue samples in red. Axes show the first and second coordinates with the percent variation explained by these coordinates. (**B**) PCoA plot of tissue samples, colored by the sample site: healthy adjacent (blue), tumor luminal surface (green), and tumor core (red). (**C**) Phylum level taxonomic summary plot showing relative abundances of major phyla present in fecal and tissue samples. Phyla that were significantly differentially abundant between feces and the three tissue sites are indicated with asterisks (** q < 0.01, *** q < 0.001).

The microbiome of feces and tissue samples was assessed by 16S rRNA gene sequencing. Fecal samples showed no significant differences in microbial richness compared to tissue samples as measured by the number of detected amplicon sequence variants (ASVs) (*p* = 0.42) and a non-significant trend towards increased microbial alpha diversity as measured by the Shannon index (*p* = 0.08). Microbial composition was compared across samples using Bray–Curtis dissimilarity, demonstrating a highly significant difference (*p* < 10^−5^) between feces and tissue (Figure 1A). This corresponded to large shifts in taxonomic composition that were evident at the phylum level. The tissue samples showed enrichment of Fusobacteriota (log2 fold change (log2FC) 1.42), Proteobacteria (log2FC 1.29), and Verrucomicrobia (log2FC 1.11) and depletion of Firmicutes (log2FC −0.42) relative to feces (Figure 1C).

Comparison across the three tissue sampling sites demonstrated no significant differences in microbial alpha diversity (*p* = 0.48 for ASV richness, *p* = 0.75 for Shannon index) or in composition (*p* = 0.62) (Figure 1B). There were no significantly differentially abundant taxa across the three sampling sites at the phylum, genus, and ASV levels. We further compared tumor tissue (core and surface) to healthy adjacent tissue and found no significant differences in alpha diversity (*p* = 0.33 for ASV richness, *p* = 0.44 for Shannon index) or composition (*p* = 0.19). There were no significant differences in phylum abundances, but at the genus level *Agathobacter* (log2FC −0.42) was reduced in the tumor samples and at the ASV level two ASVs annotated as *Actinomyces odontolyticus* (log2FC −1.2) and *Alistipes finegoldii* (log2FC −0.37) were reduced in tumor samples.

### 3.2. Tissue and Fecal Microbiota Are Associated with Mesenteric Lymph Node Involvement

The majority of subjects had progressed to locally advanced colorectal cancer (T3, 56%; T4, 6%). Most of the remaining subjects had T2 CRC (T0, 3%; T1, 12%; T2, 24%). We assessed for differences in microbial composition and diversity by tumor extent, dividing the cohort into those with locally advanced CRC (T3, T4) and those with more limited disease (T0, T1, T2). Feces and tissue samples were analyzed separately given how distinct they were from one another, while tissue samples were analyzed together with adjustment for sample site. Of the 13 subjects with tissue samples, 8 had advanced disease and 5 had more limited disease. Tissue microbiota showed significant differences in microbial composition in advanced vs. early tumor stage (*p* = 0.03) but no differences in alpha diversity metrics (Table 2 and Table 3). No taxa at the ASV, genus, or phylum levels were significantly differentially abundant between tumor extent categories. When considering each of the three tissue sites separately, none individually showed a significant association of overall microbial composition or abundances of individual microbial taxa with tumor extent (Table 4). There was no significant association of fecal alpha diversity, fecal microbial composition by beta diversity analysis, or relative abundances of individual fecal microbes with tumor extent (Table 3 and Table 4).

**Figure 2:**
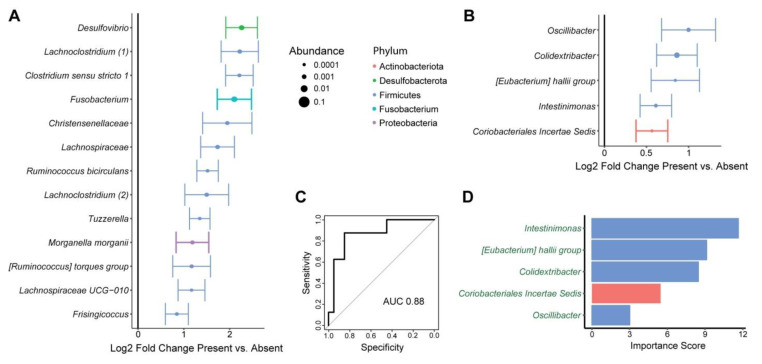
Tissue and fecal microbes are associated with mesenteric lymph node involvement by tumor. (**A**) ASVs that were differentially abundant between tissue from subjects with presence or absence of mesenteric lymph node involvement are shown. This was based upon multivariate models incorporating sample site as a covariate and subject as a random effect. Log2 fold change ± standard error is shown, with dot size proportional to mean relative abundance of the ASV. Color represents phylum. (**B**) ASVs that were differentially abundant in the feces of subjects with presence or absence of mesenteric lymph node involvement. (**C**,**D**) Random forests classifiers predicting mesenteric lymph node involvement based upon fecal microbiota. (**C**) Classifier performance is shown by a receiver operating characteristic curve with the indicated area under the curve (AUC). (**D**) Microbes included in the classifier are shown with their importance scores, which reflect contribution of the microbes to classifier accuracy. Names are colored in green to indicate that the microbes were enriched in subjects with mesenteric lymph node involvement.

Given the preclinical literature indicating that microbiota can influence tumor metastasis, we then assessed for a relationship of the microbiome with tumor spread from the primary site to the mesenteric lymph nodes [26]. In this cohort, 29% of subjects were found to have mesenteric lymph node (MLN) involvement (Table 1). MLN involvement was not significantly associated with alpha diversity metrics but was significantly associated with overall microbial composition in tissue samples (*p* = 0.025) (Table 2 and Table 3). Differential abundance testing demonstrated enrichment of 13 ASVs in tissue samples of subjects with MLN involvement compared to those without (Figure 2A). Notably, this included a member of the *Fusobacterium* genus, which has been implicated in CRC progression in preclinical and human studies. Other enriched ASVs included members of the *Desulfovibrio*, *Lachnoclostridium*, *Ruminococcus*, *Tuzzerella*, *Morganella*, and *Frisingiococcus* genera. In feces, MLN involvement was not significantly associated with alpha or beta diversity but was associated with differential abundance of five ASVs, which belonged to distinct genera from tissue microbes associated with MLN involvement (Figure 2B). Given the ready clinical accessibility of feces pre-operatively, we then asked whether these patterns could be used to predict MLN involvement from fecal microbial profiles. A random forest classifier with high accuracy for identifying subjects with MLN involvement was created (area under the receiver operating characteristics curve of 0.88) (Figure 2C). The ASV contributing most to classifier accuracy belonged to the *Intestinimonas* genus, followed by ASVs belonging to the *[Eubacterium] hallii* group and the *Colidextribacter* genera (Figure 2D).

### 3.3. Tumor T Cell Infiltration Is Associated with Tumor and Fecal Microbiota

**Figure 3:**
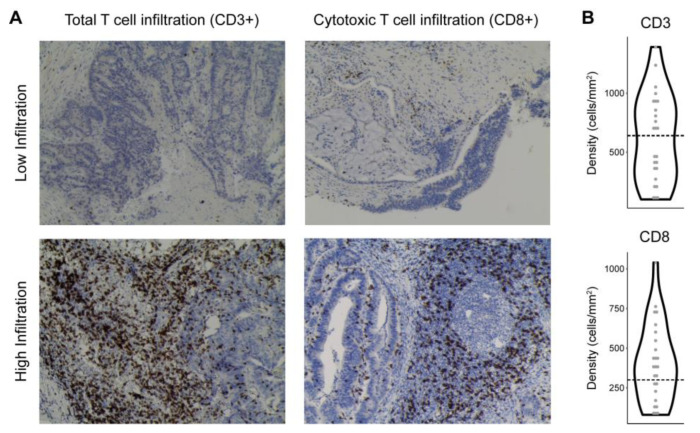
Patterns of T cell infiltration into colorectal tumor. (**A**) Representative immunohistochemistry images of CD3 (total T cells) and CD8 (cytotoxic T cells) staining in CRC samples with low or high T cell infiltration. (**B**) Violin plots showing the distribution of CD3 and CD8 density (cells/mm^2^ tumor tissue) across CRC subjects. Thresholds distinguishing low vs. high T cell infiltration are indicated by dashed lines.

To investigate T cell infiltration into tumor, slides of the tumor core were obtained from pathology specimens surgically removed from patients. Immunohistochemistry was performed to stain for CD3 (a marker of all T cells) and CD8 (a marker of cytotoxic T cells) and the number of infiltrating T cells was quantified (Figure 3). T cell infiltration data were available for 25 subjects, who were divided into low or high T cell infiltration based upon previously published cutoffs that predicted prognosis for patients with locally advanced CRC [21]. In some cases, rectal cancer patients receiving radiation pre-operatively had inadequate remaining tumor tissue for this analysis to be performed.

**Figure 4:**
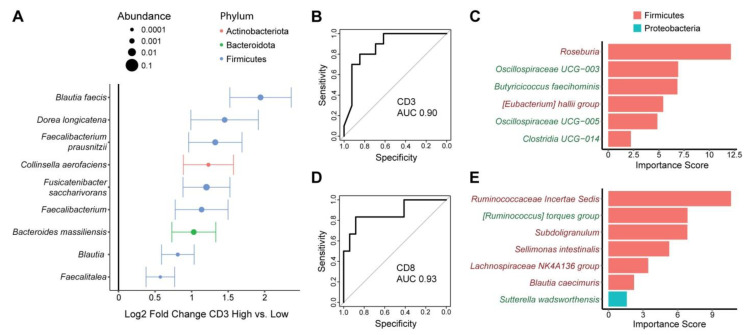
Tumor and fecal microbes are associated with tumor T cell infiltration. (**A**) ASVs present in tumor core tissue that were differentially abundant between tumors with high vs. low CD3+ T cell infiltration are shown. Effect size is represented as log2 fold change ± standard error, with dot size proportional to mean relative abundance of the ASV. Color represents phylum. (**B**–**E**) Random forests classifiers that predict high vs. low CD3+ (**B**,**C**) or CD8+ (**D**,**E**) T cell infiltration into tumors using fecal microbiota. Classifier performance is shown by receiver operating characteristic curves with area under the curve (AUC). Microbes included in the CD3 and CD8 classifiers are shown in (**C**) and (**E**), respectively, with their importance scores. Names colored in green were enriched in subjects with high T cell infiltration and names colored in red were depleted.

Tissue microbiota overall composition, but not alpha diversity, showed a significant association with CD3+ T cell and CD8+ T cell infiltration (*p* = 0.015, *p* = 0.009, respectively) (Table 2 and Table 3). Differential abundance testing demonstrated significant enrichment of a single ASV, annotated as *Blautia faecis,* in tissue samples from subjects with high CD3+ T cell infiltration (log2FC 1.91). There were no differentially abundant taxa at the genus or phylum levels with CD3+ T cell infiltration. To investigate whether specific tissue sites would show stronger association with CD3+ T cell infiltration, we performed beta diversity analysis for each site separately. There was a trend towards an association of CD3+ T cell infiltration with microbial composition in the tumor core (*p* = 0.089) but not the other sites (Table 4). Tumor core was biologically plausible as having the strongest microbiome association since T cell infiltration was measured in the tumor core. We therefore assessed for differential abundance of microbes in tumor core tissue in high vs. low CD3+ T cell infiltration. Nine ASVs were enriched in tumor core in subjects with high vs. low CD3+ T cell infiltration, including two members of the *Blautia* genus, two members of the *Faecalibacterium* genus, an ASV in the *Faecalitea* genus, and ASVs annotated as *Dorea longicatenia, Collinsella aerofaciens, Fusicatenibacter saccharivorans,* and *Bacteroides massiliensis* (Figure 4A). There were no differentially abundant taxa associated with CD8+ T cell infiltration in tissue when including all sample sites. There was no trend for beta diversity association of any of the three individual sites, but differential abundance testing of each demonstrated enrichment of *Fusicatenibacter saccharivorans* in tumor surface samples from subjects with high CD8+ T cell infiltration (log2FC 2.0) (Table 4).

We then assessed for relationships of the fecal microbiota with tumor T cell infiltration. There were no significant associations of fecal alpha or beta diversity with either CD3+ or CD8+ T cell infiltration (Table 2 and Table 3). Moreover, there were no differentially abundant ASVs associated with either T cell type after adjustment for multiple hypothesis correction. However, as there were nominally significant differentially abundant ASVs, we investigated whether they could be used to construct classifiers to predict high vs. low tumor T cell infiltration from fecal samples. We found that 6–7 ASVs were sufficient to construct random forest classifiers with high accuracy to differentiate subjects with high vs. low CD3+ and CD8+ T cell infiltration, with AUC of 0.90 and 0.93, respectively (Figure 4B,D). Different fecal microbes were included in the classifiers for each T cell type. Depletion of an ASV in the *Roseburia* genus made the strongest contribution to prediction of high CD3+ T cell infiltration (Figure 4C). This phenotype was also associated with increased abundance of two ASVs within the *Oscillospiraceae* family, one ASV in the Clostridia order, and an ASV identified as *Butyricicoccus faecihominis* as well as reduced levels of an ASV in the *[Eubacterium] hallii* group. Depletion of an ASV in the Ruminococcaceae family made the strongest contribution to classifier accuracy for predicting CD8+ T cell infiltration (Figure 4E). Subjects with high CD8+ T cell infiltration also had depletion of four other ASVs (*Subdoligranulum, Sellimonas intestinalis*, Lachnospiraceae NK4A136 group, *Blautia caecimuris*) as well as enrichment of an ASV in the *[Ruminococcus] torques* group and an ASV annotated as *Sutterella wadsworthensis.*

## 4. Discussion

This study contributes to the growing body of evidence supporting a role of the microbiota in regulating T cell trafficking and prognosis in colorectal cancer. We found that both fecal and tissue microbial profiles were associated with tumor T cell infiltration and mesenteric lymph node involvement despite highly significant differences between these two sample types. Tissue showed enrichment of Fusobacteriota, Proteobacteria, and Verrucomicrobia and depletion of Firmicutes compared to feces. This is consistent with a prior small study, which found numerically increased Proteobacteria and decreased Firmicutes in mucosal vs. fecal samples from six CRC patients [36]. Interestingly, no significant differences were found among the three tissue sites, which included tumor core, tumor surface, and healthy adjacent tissue. Several microbes were differentially abundant when comparing the two tumor sites to healthy adjacent tissue, though these taxa did not match those seen in a prior study comparing tumor microbiota to healthy adjacent mucosa [26]. 

Of the different sampling sites, tumor core microbiota showed the strongest association with degree of total T cell infiltration in CRC patients. Several of the tumor core microbes associated with high T cell infiltration have been previously linked to improved immunotherapy response. High levels of *Faecalibacterium* have been associated with significantly prolonged progression-free survival in patients undergoing anti-PD-1 immunotherapy for melanoma [19,37]. *Collinsella aerofaciens* was more abundant in responders to anti-PD-1 immunotherapy for melanoma [38]. Similarly, *Bacteriodes massiliensis* was associated with longer progression-free survival of melanoma patients receiving immune checkpoint inhibitory therapy [39]. The relationship of these microbes with improved outcomes of immune checkpoint inhibitory therapy suggests a role for these microbes in regulating anti-tumor T cell responses. 

The tumor core microbial signature of CRC patients with high T cell infiltration also included multiple microbes known for short chain fatty acid (SCFA) production including members of the *Blautia*, *Faecalibacterium*, and *Dorea* genera [40]. *Blautia* is believed to be protective in a wide range of disease states, with reduced abundance in CRC patients as well as inflammatory bowel disease, liver cirrhosis, and obesity [41,42,43,44,45]. *Faecalibacterium prausnitzii (F. prausnitzii)* is known for its anti-inflammatory effect and may have a protective role in Crohn’s disease and CRC [46,47,48,49]. *Dorea longicatena* has been associated with maintenance of remission in Crohn’s disease [50]. A common mechanism for the disease-protective effects of these microbes is their production of butyrate, a SCFA with immunomodulatory effects that has recently been reported in preclinical models to promote the long term survival and anti-tumor activity of cytotoxic T cells [51,52].

Although the fecal microbiota overall was less strongly associated with increased T cell infiltration than tumor microbiota, relative abundances of specific fecal microbes were sufficient to train classifiers with high accuracy for differentiating subjects with high vs. low total or cytotoxic T cell infiltration. The fecal microbes that contributed to classifier accuracy were distinct from these that were enriched in the tumor microbiota of CRC patients with high T cell infiltration. Patients with high total and cytotoxic T cell infiltration showed a mix of enrichment of butyrate producers such as *Butyricicoccus faecihominis* and a member of the *[Ruminococcus] torque* group and reduction in other butyrate producers including *Roseburia*, *Subdoligranulum*, and *Blautia caecimuris*. Intestinal microbes outside of the tumor microenvironment could conceivably influence T cell recruitment to tumors through effects on trafficking of mucosal immune cells or cross-talk with the tumor-associated microbiome [53]. 

We further found that the fecal and tissue microbiota of CRC were associated with mesenteric lymph node involvement. This is in line with existing preclinical and human data indicating that the microbiome modulates metastatic potential of CRC cells [25]. *Fusobacterium* species have been of particular interest as potential drivers of metastasis. Primary and matched liver metastases have been shown to harbor the same *Fusobacterium* strains, and *Fusobacterium* was found to persist in CRC xenografts and promote tumor growth [24]. Another study reported that metastatic CRC patients have increased tumor *Fusobacterium* compared to non-metastatic CRC patients [54]. The authors also found that *Fusobacterium* was detected in a higher proportion of mesenteric lymph nodes with CRC metastasis than those without metastasis. Consistent with this prior literature, we found that *Fusobacterium* was enriched in the tissue microbiota of CRC patients with mesenteric lymph node involvement compared to those without. We also found an association of *Desulfovibrio* in the tissue microbiota with mesenteric lymph node involvement. This is consistent with a study that reported significantly increased *Desulfovibrio* in the fecal microbiota of CRC patients with liver metastasis, though we did not find the same association in fecal samples of our cohort [55]. We identified additional microbes that have not been previously reported to be associated with mesenteric lymph node involvement. Among these, we notably observed enrichment of *Morganella morganii*. This species has recently been reported to produce indolamines, which have genotoxic properties and could promote mutations that facilitate CRC metastasis [56].

Our study has several limitations including small sample size, particularly for tissue samples, and left-sided predominance of CRC cases, which limits generalizability of our results to CRC of the right colon. Also, fecal samples were collected by the subjects in their homes, which introduces greater risk of sample handling issues compared to collection at clinical sites. Additionally, our study does not have a validation cohort. The taxa of tumor core microbiota associated with high CD3 T cell infiltration did not overlap with those reported to differentiate the tumor microbiota of high vs. low CD3 T cell infiltration in a prior study, perhaps reflecting the small sample sizes in each study and differences in patient populations [23]. This demonstrates the need for larger follow-up studies incorporating phenotypically diverse CRC populations to identify microbial drivers of tumor T cell infiltration. Moreover, the microbiome was assessed by 16S rRNA gene sequencing, which does not consistently achieve species level taxonomic resolution and does not directly assess microbial function. 

Despite these limitations, this study provides valuable supportive evidence that the tumor-associated and fecal microbiota may modulate CRC phenotype including mesenteric lymph node metastasis and extent of T cell infiltration. Classifiers were created to predict mesenteric lymph node involvement and the degree of T cell infiltration from pre-operative fecal microbial profiles. This demonstrates the potential to develop non-invasive microbial biomarkers for pre-operative risk stratification of CRC patients. The relationship of the microbiome with tumor T cell infiltration supports the possibility of therapeutically modulating the microbiome to promote anti-tumor immunity following surgical resection to reduce risk of recurrence.

## Figures and Tables

**Table 1:** Cohort demographics.

	N	%
Age, years, median (IQR)	56.0 (49.3, 64.0)	
Sex		
Male	21	61.8%
Female	13	38.2%
Tumor Location		
Ascending colon	1	2.9%
Transverse colon	4	11.8%
Sigmoid colon	13	38.2%
Rectosigmoid junction	5	14.7%
Rectum only	11	32.4%
Tumor Stage		
T0	1	2.9%
T1	4	11.8%
T2	6	23.5%
T3	19	55.9%
T4	2	5.9%
Lymphovascular Invasion		
Yes	7	20.6%
No	27	79.4%
Mesenteric Lymph Node Involvement		
Yes	10	29.4%
No	24	70.6%

**Table 2:** Fecal and tissue microbiota alpha diversity associations (*p*-values) with tumor extent, mesenteric lymph nodes, and T cell infiltration.

	Tissue		Feces	
	ASV Richness	Shannon Index	ASV Richness	Shannon Index
Age	0.164	0.073	0.248	0.198
Sex	0.683	0.940	0.970	0.739
Tumor extent	0.457	0.917	0.394	0.202
Mesenteric nodes	0.298	0.766	0.510	0.339
CD3 infiltration	0.596	0.472	0.890	0.824
CD8 infiltration	0.698	0.878	0.796	0.614

**Table 3:** Fecal and tissue microbiota beta diversity associations (*p*-values and R^2^) with tumor extent, mesenteric lymph nodes, and T cell infiltration. Significant *p*-values (<0.05) are indicated in bold.

	Tissue		Feces	
	R^2^	*p*-Value	R^2^	*p*-Value
Age	0.038	**0.024**	0.037	0.437
Sex	0.045	**0.027**	0.024	0.953
Tumor extent	0.044	**0.030**	0.035	0.533
Mesenteric nodes	0.050	**0.025**	0.043	0.222
CD3 infiltration	0.060	**0.015**	0.032	0.902
CD8 infiltration	0.043	**0.009**	0.035	0.807

**Table 4:** Beta diversity associations of individual tissue regions with tumor stage, mesenteric lymph nodes, and T cell infiltration.

	Tumor Core		Tumor Surface		Healthy Adjacent	
	R^2^	*p*-Value	R^2^	*p*-Value	R^2^	*p*-Value
Tumor stage	0.086	0.397	0.108	0.142	0.094	0.231
Mesenteric nodes	0.087	0.441	0.103	0.139	0.064	0.942
CD3+ infiltration	0.112	*0.089*	0.065	0.774	0.081	0.513
CD8+ infiltration	0.055	0.957	0.062	0.809	0.065	0.888

## Data Availability

The 16S rRNA gene sequencing data are available from NCBI Bioproject, PRJNA909427.
